# A novel fluorescent assay for sucrose transporters

**DOI:** 10.1186/1746-4811-8-13

**Published:** 2012-04-04

**Authors:** Peter J Gora, Anke Reinders, John M Ward

**Affiliations:** 1 Department of Plant Biology, University of Minnesota, 250 Biological Sciences Center, 1445 Gortner Ave., St. Paul, MN 55108-1095, USA

## Abstract

**Background:**

We have developed a novel assay based on the ability of type I sucrose uptake transporters (SUTs) to transport the fluorescent coumarin β-glucoside, esculin. Budding yeast (*Saccharomyces cerevisiae*) is routinely used for the heterologous expression of SUTs and does not take up esculin.

**Results:**

When type I sucrose transporters StSUT1 from potato or AtSUC2 from Arabidopsis were expressed in yeast, the cells were able to take up esculin and became brightly fluorescent. We tested a variety of incubation times, esculin concentrations, and buffer pH values and found that for these transporters, a 1 hr incubation at 0.1 to 1 mM esculin at pH 4.0 produced fluorescent cells that were easily distinguished from vector controls. Esculin uptake was assayed by several methods including fluorescence microscopy, spectrofluorometry and fluorescence-activiated cell sorting (FACS). Expression of the type II sucrose transporter OsSUT1 from rice did not result in increased esculin uptake under any conditions tested. Results were reproduced successfully in two distinct yeast strains, SEY6210 (an invertase mutant) and BY4742.

**Conclusions:**

The esculin uptake assay is rapid and sensitive and should be generally useful for preliminary tests of sucrose transporter function by heterologous expression in yeast. This assay is also suitable for selection of yeast showing esculin uptake activity using FACS.

## Background

Sucrose transporters (SUTs or SUCs) play a critical role in long distance transport of carbohydrates in plants. Products of photosynthesis must have an efficient means of being distributed to cells in the plant that depend on the net import of fixed carbon such as in roots, flowers, and seeds. In many plants, this is achieved by active loading of the phloem using H^+^-coupled sucrose transporters [1]. The first sucrose uptake transporter (SUT) was cloned from spinach by expression in the yeast strain SuSy7 [2]. SuSy7 is an invertase mutant that expresses plant sucrose synthase in the cytoplasm. Growth of SuSy7 on sucrose depends on expression of a sucrose uptake transporter. Growth assays using SuSy7 have been subsequently used to demonstrate sucrose transport activity of many cloned SUT homologs such as AtSUT4 [3], OsSUT1 and OsSUT3 [4], TaSUT1 [5], and VvSUC27 [6]. The SuSy7 growth assay is rapid and does not require special equipment; however, SuSy7 vector controls do grow slowly on sucrose media making it difficult to distinguish low sucrose transporter activity from background.

Here we introduce a novel assay for sucrose transporter activity based on the ability of type I SUTs to transport the highly fluorescent molecule esculin (6,7-dihydroxycoumarin β-D-glucoside). The type I SUTs AtSUC2 and AtSUC9 transport the fluorescent β-glucosides esculin and fraxin (7,8-dihydroxy-6-methoxy-coumarin-8-β-D-glucoside) at a rate similar to that of sucrose [7,8] while type II SUTs HvSUT1, ShSUT1, OsSUT1 and OsSUT5 do not transport esculin or fraxin [7,9]. Type III SUTs are vacuolar and, in general, have a wide substrate specificity similar to type I SUTs [8]. We have analyzed the substrate specificity of one type III SUT, LjSUT4 from *Lotus japonicus*, and it does not transport esculin or fraxin [10].

Similar to the SuSy7 growth assay, the method presented here involves expression of plant SUT cDNAs in budding yeast, *Saccharomyces cerevisiae*. Yeast expressing type I SUTs accumulate esculin or fraxin and become highly fluorescent. Esculin shows an excitation peak at 367 nm and emits in the visible region at 454 nm and fluorescent cells can easily be detected by fluorescence microscopy or using a fluorescence plate reader. Untransformed yeast do not accumulate esculin and therefore do not become fluorescent.

## Results

**Figure 1:**
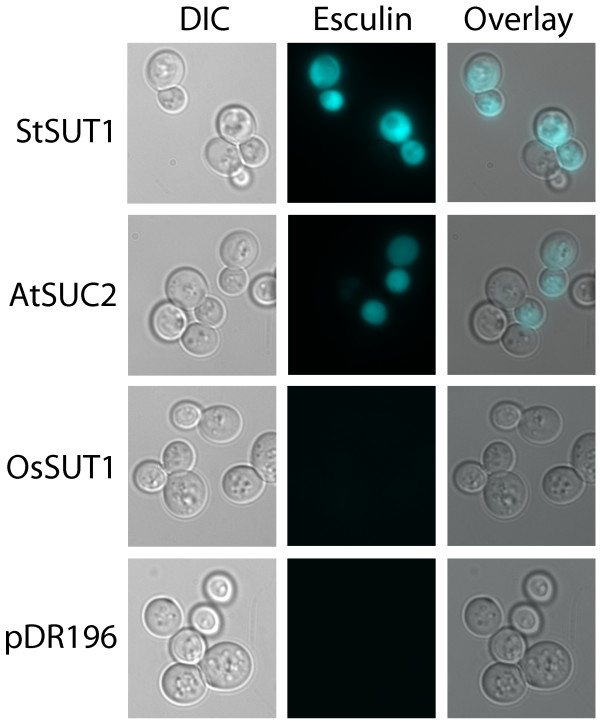
**Esculin uptake by yeast expressing sucrose transporter cDNAs**. Yeast (SEY6210) transformed with plant sucrose transporters StSUT1, AtSUC2, OsSUT1 or vector control (pDR196), indicated on the left, were incubated for one hour in 1 mM esculin in 25 mM sodium phosphate buffer (pH 4.0). The cells were washed and visualized at 1000× magnification using differential contrast (DIC, column 1) or fluorescence microscopy (column 2). For fluorescence microscopy the following filters were used: excitation filter 426-446 nm, 455 nm LP dichroic mirror, 460-500 nm emission filter. The DIC and fluorescence images were overlaid as shown in column 3.

Coumarins are brightly fluorescent and useful for labelling cells for fluorescence microscopy for example [11]. Type I SUTs transport the plant coumarin glucosides fraxin and esculin [7] whereas yeast strain SEY6210 does not, as indicated by the lack of fluorescence of the vector control (pDR196) in Figure 1. Yeast expressing the type I StSUT1 from potato or AtSUC2 (At1g22710) from Arabidopsis became brightly fluorescent when incubated with esculin (Figure 1). Consistent with previous analysis of the substrate specificity of OsSUT1 (Os03g07480) from rice [12], yeast expressing OsSUT1 did not show higher fluorescence than vector controls. Type II SUTs are more selective for sucrose than type I SUTs [8] and it has been shown that type II SUTs HvSUT1 from barley and ShSUT1 from sugarcane do not transport fraxin or esculin [7].

**Figure 2:**
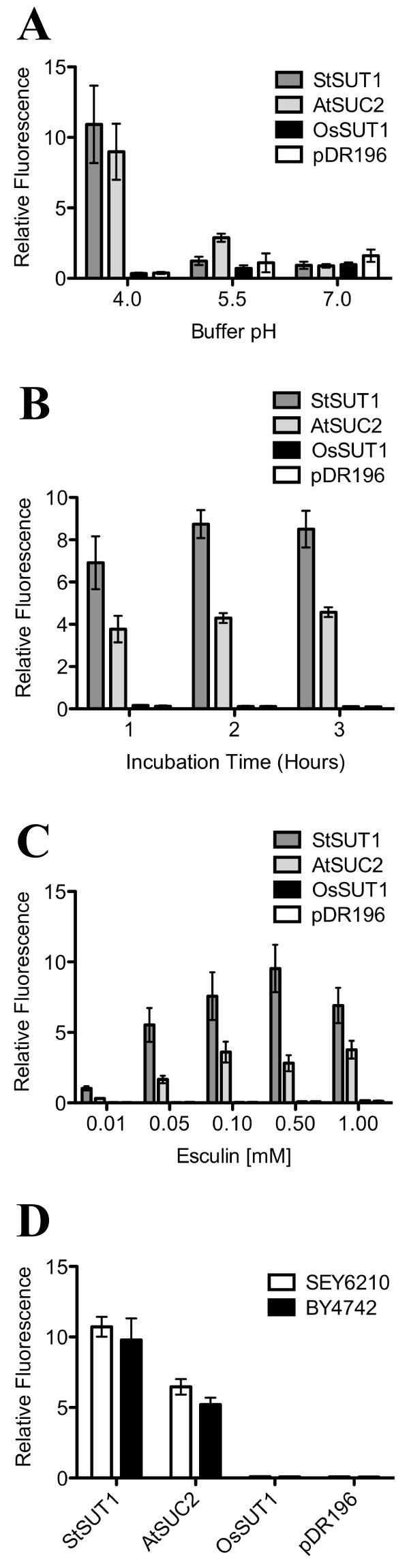
**Analysis of esculin uptake by yeast expressing plant sucrose transporters**. Yeast (SEY6210, except where indicated) transformed with sucrose transporter cDNAs StSUT1, AtSUC2, OsSUT1 or empty vector (pDR196) were incubated in 25 mM sodium phosphate buffer containing the fluorescent coumarin glucoside esculin. The cells were washed and fluorescence was determined at 367 nm excitation and 454 nm emission using a fluorescence plate reader. Cell density was determined by absorbance at 600 nm. Values are means for each treatment, normalized for cell density, ± SE (n = 4). (A) Effect of pH on esculin uptake. Yeast cells were incubated for 1 hr with 1 mM esculin at three pH values. (B) Effect of incubation time on esculin uptake. Yeast cells were incubated with 1 mM esculin at pH 4.0 for the indicated times. (C) Concentration dependence of esculin uptake. Yeast cells were incubated with different concentrations of esculin at pH 4.0 for 1 hr. (D) Esculin uptake by two yeast strains, SEY6210 and BY4742, transformed with sucrose transporter cDNAs. Yeast cells were incubated in 1 mM esculin at pH 4.0 for 1 hr.

To determine whether the uptake of the coumarin glucoside esculin into yeast could serve as a useful assay for sucrose transporter activity, we tested a number of incubation conditions. Yeast expressing StSUT1 and AtSUC2 accumulated esculin at pH 4.0 to a much greater extent than at pH 5.5 or pH 7.0 (Figure 2A). This is consistent with the pH dependence of these transporters for sucrose uptake [13,14]. Yeast expressing OsSUT1 did not show fluorescence above the vector control at any pH tested.

Different times of incubation of the yeast cells with 1 mM esculin at pH 4.0 were tested. Results were similar for 1, 2, or 3 hr incubations. Yeast cells expressing StSUT1 or AtSUC2 showed high fluorescence while yeast expressing OsSUT1 were similar to vector controls (Figure 2B). Concentrations of esculin from 0.01 to 1.0 mM at pH 4.0 were tested. After 1 hr incubation, fluorescence of yeast cells expressing StSUT1 increased up to approximately 0.5 mM and for AtSUC2 up to 0.1 mM esculin (Figure 2C). Concentrations of esculin as low as 0.05 mM were effective for this assay but a higher concentration may be desirable in case the SUT of interest has unknown or relatively low activity or a high *K_m_*.

The yeast strain SEY6210 [15] is routinely used for ^14^C-sucrose uptake assays for example [16] because it is an invertase mutant. We tested whether another yeast strain, BY4742 [17], would also be suitable for this assay. Results produced with BY4742 were almost identical to those observed with SEY6210 (Figure 2D) suggesting that multiple yeast strains are compatible with this assay, including invertase wild-type strains.

**Figure 3:**
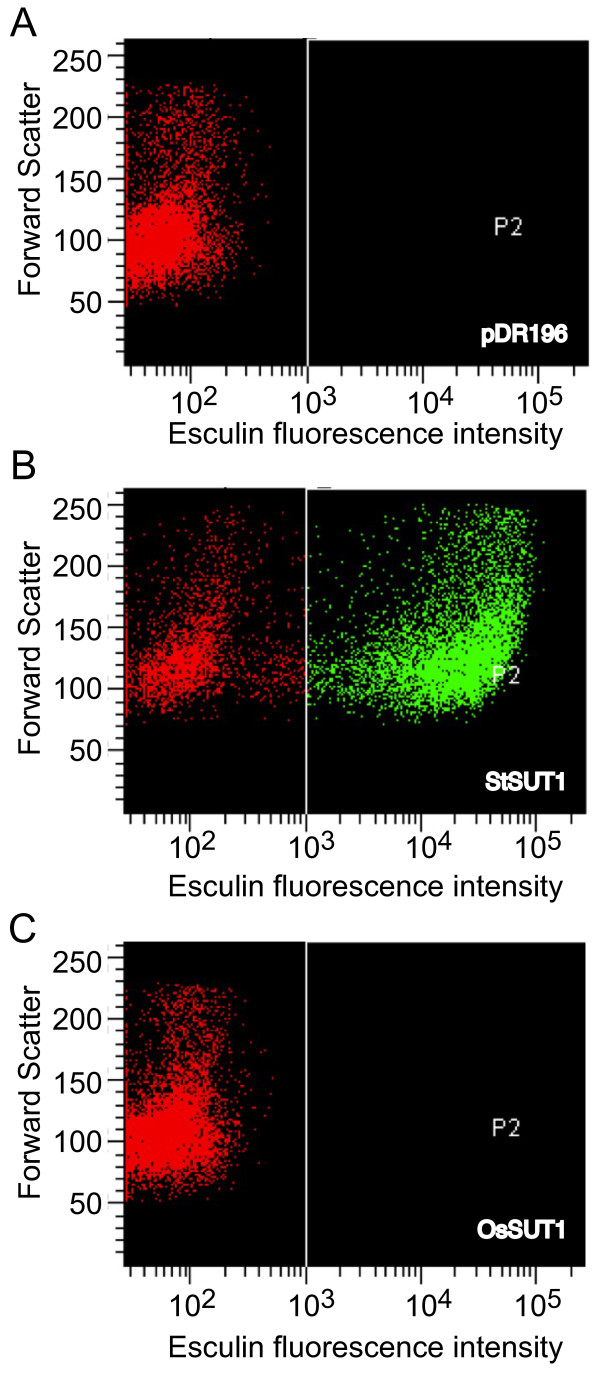
**Esculin uptake by yeast cells expressing StSUT1 was detected using FACS**. Yeast cells (SEY6210) were incubated in 1 mM esculin in 25 mM Na-phosphate buffer (pH 4) for 3 hr at 30°C with shaking. Cells were collected by centrifugation, washed with 25 mM Na-phosphate buffer (pH 4.0) and resuspended with the same buffer at a density of 10^7 ^cells/ml. Cells were identified based on forward scatter (relative cell size) vs. esculin intensity. Fluorescent cells were detected by flow cytometry using a BD Biosciences FACSVantage DiVa sorter with a UV laser, excitation 355 nm, and a 450/50 detection filter (A) pDR196 vector control; (B) StSUT1 in pDR196); (C) OsSUT1 in pDR196).

Yeast cells that accumulated esculin could also be detected using FACS. SEY6210 cells transformed with either StSUT1, OsSUT1, or the empty pDR196 vector were incubated in 1 mM esculin at pH 4.0 for 3 hrs, washed, and resuspended to a density of 10^7 ^cells/ml. Fluorescent cells were detected using a UV laser and 350 nm excitation and 450/50 nm emission filters. Results are shown in Figure 3. Yeast transformed with the empty vector (Figure 3A) showed low esculin fluorescence (X axis). Approximately 60% of cells carrying the StSUT1 plasmid (Figure 3B) showed high esculin fluorescence consistent with the results shown in Figure 1 and 2. Consistent with the inability of OsSUT1 to transport esculin, yeast transformed with OsSUT1 (Figure 3C) were not more fluorescent than vector controls. These results indicate that single yeast cells expressing an esculin transporting activity can easily be selected by FACS.

## Discussion

Several different assays for sucrose transporter activity have been used. The earliest studies measured ^14 ^C-sucrose uptake into the vascular tissue of source leaves or leaf discs [18]. Isolated protoplasts were used to study the basic properties of cellular sucrose uptake, such as the dependence on a transmembrane H^+ ^gradient [19]. Plasma membrane vesicles provided the first cell-free system for studying sucrose transporters [20]. Once the first SUT was cloned [2], heterologous expression in yeast proved to be a powerful tool to study individual transporters. Both ^14^C-sucrose uptake into yeast and growth of SuSy7 on sucrose media continue to be used in the study of cloned SUTs. Expression in *Xenopus *oocytes and electrophysiology [21] is used to study SUT kinetics and substrate specificity. Patch clamp analysis of *Xenopus *oocyte plasma membrane has been used to study reversal of ZmSUT1 [22]. Additionally, patch clamping of vacuole membranes was used to study a type III SUT and sucrose antiport activity [23].

One advantage of the esculin uptake assay presented here is that it is rapid and detection can be accomplished using several different methods such as fluorescence microscopy, fluorescence plate reader, or FACs. Results can also be obtained using a UV gel documentation system (not shown). Another advantage of this assay is that detection can be made at the single cell level. This will allow library screening for esculin uptake activity using FACS.

We observed that approximately 60% of the yeast transformed with StSUT1 or AtSUC2 showed strong esculin fluorescence as detected by fluorescence microscopy (Figure 1) or FACS (Figure 3). This could be due to expression from the PMA1 promoter in the pDR196 vector which is down-regulated when cells begin to enter stationary phase [24]. Due to the fact that cells are grown in a microtiter plate to a high density, it is possible that a significant portion of the cells are in or entering stationary phase when the assay is performed. PMA1 expression is also regulated during the cell cycle with significant up-regulation occurring in the G2 phase [25,26]. In addition, the rate of proteolysis of the sucrose transporters in yeast is not known. Also, esculin uptake is dependent on the electrochemical proton gradient which is influenced by other transporters in the yeast plasma membrane including the PMA1 proton pump. Changes in these activities during the cell cycle could result in the observed heterogeneity in esculin fluorescence.

The acidic pH optimum for esculin uptake (pH 4.0) is consistent with the proton-coupled mechanism of the sucrose transporters tested; both AtSUC2 and StSUT1 are known to have an acidic pH optimum for sucrose uptake. Low pH buffers may also lower the fluorescence background resulting from incomplete washing of esculin from the outside of yeast cells. From pH 4 to 7 the excitation peak of esculin shifts from 340 nm to 367 nm. Additionally, the fluorescence emission at 454 nm is roughly five times greater for esculin at pH 7.0 than at pH 4.0 so that, in theory, esculin outside the cell will not fluoresce as brightly as the esculin inside the cell which is at a cytosolic pH. This is consistent with the slightly higher background observed at pH 5.5 and 7.0 (Figure 2A).

The esculin uptake assay has several limitations. Type II SUTs such as OsSUT1 do not transport esculin or fraxin [7-9] and therefore cannot be assayed using this method. This assay is limited to type I SUTs that only are found in eudicot species [8]. This assay, as presented, is not quantitative because initial rates of uptake were not measured. To determine kinetic values such as *K*_m _or test changes in SUT activity, such as response to inhibitors, would require additional refinements to measure the rate of fluorescence increase within a few minutes of esculin addition. Also, esculin is not as soluble as sucrose, its limit of solubility is approximately 5 mM at 20°C. There are some general limitations in using yeast uptake for determining *K*_m _values for SUTs; measuring ^14 ^C sucrose uptake in yeast tends to underestmate *K*_m _due to dissipation of the membrane potential at high substrate concentrations as discussed by Chandran et al. [27]. *K*_m _can be more accurately determined using oocyte expression and electrophysiology. Esculin or fraxin may be useful as a tracer to measure phloem loading and long-distance transport in plants or to measure uptake into specific cell types. However, uptake of this substrate might not be specific for SUTs; other transporters in the major facilitator superfamily (MFS) [28] or SWEET family [29], for example, may also transport esculin. Overall, we think that the assay presented here is suitable to test whether a type I SUT is active when expressed in yeast and may have some advantages over the SuSy7 growth assay for this purpose.

## Methods

### Yeast strains and DNA constructs

The following yeast strains were used in this study: SEY6210 [MATα leu2-3, 112 ura3-52 his3-Δ200 trp1-Δ901 lys2-Δ801 suc2-Δ9] [15] and BY4742 [MATα; his3Δ1; leu2Δ0; lys2Δ0; ura3Δ0] [17]. Competent yeast cells were prepared and transformed as described [30] with sucrose transporter constructs in pDR196 [31] or pDR196/GW [32]. The AtSUC2 ORF in pCR2.1 [27] was excised using EcoRI and cloned into the same site of pDR196. The following plasmids used in this study were previously published: StSUT1 in pDR196 [33] and OsSUT1 in pDR196/GW [12].

### Microtiter plate inoculation

Microtiter plates containing 200 μl per well of liquid SD-URA media (1.7 g/l yeast nitrogen base without amino acids and ammonium sulfate (Difco), 5 g/l ammonium sulfate, 20 g/l glucose, 30 mg/l tryptophan, 30 mg/l lysine, 60 mg/l leucine, 30 mg/l histidine, and 60 mg/l adenine hemisulfate) were inoculated with yeast transformants. Four independent transformants were used for each construct. The plates were sealed and placed in a 30°C shaker overnight (approximately 16 hrs).

### Sucrose transporter assay using fluorescent esculin substrate

1. To collect the yeast cells, microtiter plates were centrifuged in a GH 3.7, 160 mm rotor at 2700 rpm (1.3 kxg) for five minutes after which the SD-URA supernatant was aspirated.

2. Esculin (1 mM or concentration indicated in figure legends) in 200 μl of phosphate buffer (25 mM Na_2_HPO_4_, pH 4 or pH indicated in the figure legends adjusted with phosphoric acid) was added to each well.

3. The plates were sealed and the yeast re-suspended by vortexing for 30 sec. The plates were then incubated at 30°C for one hour (or time indicated in the figure legends) with shaking.

4. Microtiter plates were centrifuged at 1.3 kxg for five minutes and the supernatant was aspirated.

5. The cells were washed by adding 200 μl of phosphate buffer of the same pH to each well.

6. The microtiter plate was re-sealed, and the yeast cells were re-suspended by vortexing for 30 sec.

7. The microtiter plate was centrifuged at 1.3 kxg for five minutes and the supernatant was aspirated.

8. 200 μl of phosphate buffer of the same pH was added to each well and the cells were re-suspended by gently pipetting up and down.

9. The contents of the microtiter plate were then transferred to a new, black microtiter plate and fluorescence was read using a BioTek^® ^SYNERGYMx spectrofluorometer at 367 nm excitation and 454 nm emission.

10. A second microtiter plate to determine the OD_600 _of the cells was prepared by adding 150 μl of ddH_2_O and 50 μl of the cell suspensions from the black microtiter plate to each well. Water was used as a blank.

11. Cell density was obtained by measuring OD_600 _on a BioTek^® ^PowerWave 340 plate reader. The measurements were adjusted to compensate for the 1:4 dilution used.

12. Fluorescence per unit OD_600 _was calculated to determine relative fluorescence.

### Preparation of slides and DIC/Fluorescence microscopy

Yeast cells (20 μl of the cell suspension) from Step 8 (above) were immobilized on a coverslip coated with 0.1% polyethyleneimine (Sigma). The coverslip was then placed on a glass slide and sealed with wax to prevent dehydration. The cells were visualized with a Nikon E800 microscope using differential contrast (DIC) and fluorescence microscopy. A Nikon CFP fluorescence cube was used for fluorescence microscopy (excitation filter 426-446 nm, 455 nm LP dichroic mirror, 460-500 nm emission filter). Note that the filters used for microscopy were not optimal for esculin fluorescence.

### Fluorescence-activated cell sorting (FACS)

Yeast strain SEY6210, transformed with StSUT1, OsSUT1 or empty vector (pDR196) were grown as described above and incubated with 1 mM esculin in phosphate buffer (pH 4) with shaking at 30°C for 3 h. Cells were then centrifuged at 2700 rpm (1.3 kxg), washed with phosphate buffer and resuspended with the same buffer at a density of 10^7 ^cells/ml. Fluorescent cells were isolated using a BD Biosciences FACSVantage DiVa cell sorter (UV laser, excitation 355 nm, emission 450/50 detector configuration). PBS (pH 7) was used as running and collection buffer.

## Competing interests

The authors declare that they have no competing interests.

## Authors' contributions

The experiments were planned by AR and JMW. PJG and AR performed the experiments, analyzed the data and prepared the figures. The manuscript was written by PJG, AR and JMW. All authors read and approved the final manuscript.
